# Percutaneous transvenous coil embolization (PTCE) for treatment of single extrahepatic portosystemic shunt in dogs

**DOI:** 10.1186/s12917-023-03783-1

**Published:** 2023-10-19

**Authors:** Kumiko Ishigaki, Kazushi Asano, Kei Tamura, Naoki Sakurai, Kazuyuki Terai, Tatsuya Heishima, Orie Yoshida

**Affiliations:** Laboratory of Veterinary Surgery, Department of Veterinary Medicine, College of Bioresource Sciences, Nihon University, 1866 Kameino, Fujisawa, Kanagawa 252–0880 Japan

**Keywords:** Coil embolization, Dog, Extrahepatic, Portosystemic shunt

## Abstract

**Background:**

There is limited information regarding percutaneous transvenous coil embolization (PTCE) for single extrahepatic portosystemic shunt (PSS). This study aimed to describe the procedure and outcome of PTCE in dogs with a single extrahepatic PSS. Forty-two privately owned dogs were included in this study. All dogs were diagnosed with extrahepatic PSS by computed tomography (CT). Preoperative CT images were used to evaluate the diameter of the PSS for coil placement. A multipurpose balloon catheter was percutaneously inserted into the PSS via the jugular vein, and transvenous retrograde portography (TRP) and measurement of blood pressure in the PSS (pPSS) were performed during balloon inflation; one or more embolization coils were implanted via the catheter.

**Results:**

In most cases, preoperative median fasting and postprandial serum total bile acid (TBA) concentrations were high (fasting, 86.5 μmol/L [ 3.7–250.0 μmol/L]; postprandial, 165.5 μmol/L [ 1.5–565.0 μmol/L]). CT revealed that 30 dogs had left gastrophrenic shunt; eight had left gastroazygos shunt; and one each had left gastrocaval, splenocaval, splenophrenic, and left colocaval shunt. TRP revealed that intrahepatic portal vascularity was clearly detectable in all dogs. The median values of pPSS before and during the balloon occlusion were 4.8 mmHg [2.0–13.0 mmHg] and 8.6 mmHg [5.0–18.0 mmHg], respectively. The median number and diameter of coils used were 2 coils [1 – 5 coils] and 8.0 mm [4.0 – 12.0 mm], respectively. The median times of irradiation and PTCE were 9 min [4–26 min] and 40 min [23–75 min], respectively. The median fasting and postprandial TBAs significantly decreased to 8.2 μmol/L [0.3–45.1 μmol/L, *n* = 38*, p* = 0.0028] and 19.8 μmol/L [0.3–106.7 μmol/L, *n* = 38, *p* = 0.0018], respectively, approximately 1 month after PTCE. The clinical success rate of PTCE without requirement for a second surgery was 95.2% (40/42 dogs). During revision surgery, one dog underwent surgical ligation and, in another dog, an ameroid constrictor was placed.

**Conclusions:**

PTCE was clinically effective in treating single extrahepatic PSS in dogs. Preoperative CT and TRP prior to PTCE might be clinically valuable for choosing the size of embolization coils, deciding the appropriate location of coil implantation, and estimating the number of coils to be implanted. PTCE is a promising alternative to conventional surgical procedures for single extrahepatic PSS in dogs.

**Supplementary Information:**

The online version contains supplementary material available at 10.1186/s12917-023-03783-1.

## Background

Congenital portosystemic shunts (PSSs) are abnormal vascular communications between the portal vein and systemic venous circulation, and depending on their location, they are categorized as intrahepatic or extrahepatic PSS [1]. Surgical attenuation of the shunting vessel is the treatment of choice for congenital PSS in dogs. The technical skills required, outcome, and prognosis of the surgical attenuation depend on the type of PSS and intrahepatic portal venous vasculature. Although conventional surgical ligation is an effective treatment for canine extrahepatic PSS, complete PSS ligation is not always possible through a one-stage surgery. In previous reports, complete ligation was possible in only 17–55% of the dogs with extrahepatic PSS [2–6]. In addition, the mortality rate associated with surgical ligation is known to be 2.1–32%, and postoperative complications including post-attenuation neurological signs, hemorrhage, portal hypertension, and recurrence of clinical signs have been observed [4–8]. Thin-film banding and ameroid constrictor placement techniques have been developed for gradual attenuation of extrahepatic PSS in dogs [9–14]. However, the incidence of postoperative portal hypertension or multiple acquired PSS using these methods has been reported to be 2–14% [9, 13].

There are a few reports on the successful treatment of intrahepatic and extrahepatic PSSs by percutaneous transvenous coil embolization (PTCE) [15–22]. The results of a previous large-scale clinical study showed that endovascular attenuation of intrahepatic PSS in dogs might reduce the morbidity and mortality rates, with similar clinical success rates as conventional surgical procedures [22]. However, limited information is available regarding PTCE for single extrahepatic PSS. We hypothesized that PTCE can be an effective alternative treatment for canine extrahepatic PSS. The purpose of this study was to describe the procedure and outcome of PTCE in dogs with single extrahepatic PSS.

## Results

The study included forty-two dogs: toy poodles (*n* = 9), miniature schnauzers (*n* = 7), papillons (*n* = 4), Chihuahua (*n* = 3), Jack Russel terriers (*n* = 3), Kaninchen Dachshund (*n* = 3), Shih-Tzu (*n* = 3), Yorkshire terriers (*n* = 3), Maltese (*n* = 2), Miniature Dachshund (*n* = 2), cross breed (*n* = 2), and American Cocker Spaniel (*n* = 1). The median age was 4.4 years [0.7–11.3 years], and no gender predisposition was observed (males, *n* = 23; females, *n* = 19). The median body weight was 4.5 kg [1.4–8.8 kg]. The main clinical signs were associated with hepatic encephalopathy in 11 dogs, urolithiasis in 10 dogs, and vomiting and anorexia in 13 dogs. One dog presented with obvious hepatic encephalopathy and clinical signs associated with urolithiasis. On the other hand, there were 9 dogs that presented without obvious clinical signs. All dogs, except four in whom serum total bile acid (TBA) concentrations were not determined, showed high levels of preoperative median fasting and postprandial serum TBA: 86.5 μmol/L [3.7–250.0 μmol/L, *n* = 38] (reference, < 10 μmol/L) and 165.5 μmol/L [1.5–565.0 μmol/L, *n* = 38] (reference, < 25 μmol/L), respectively.

**Fig. 1:**
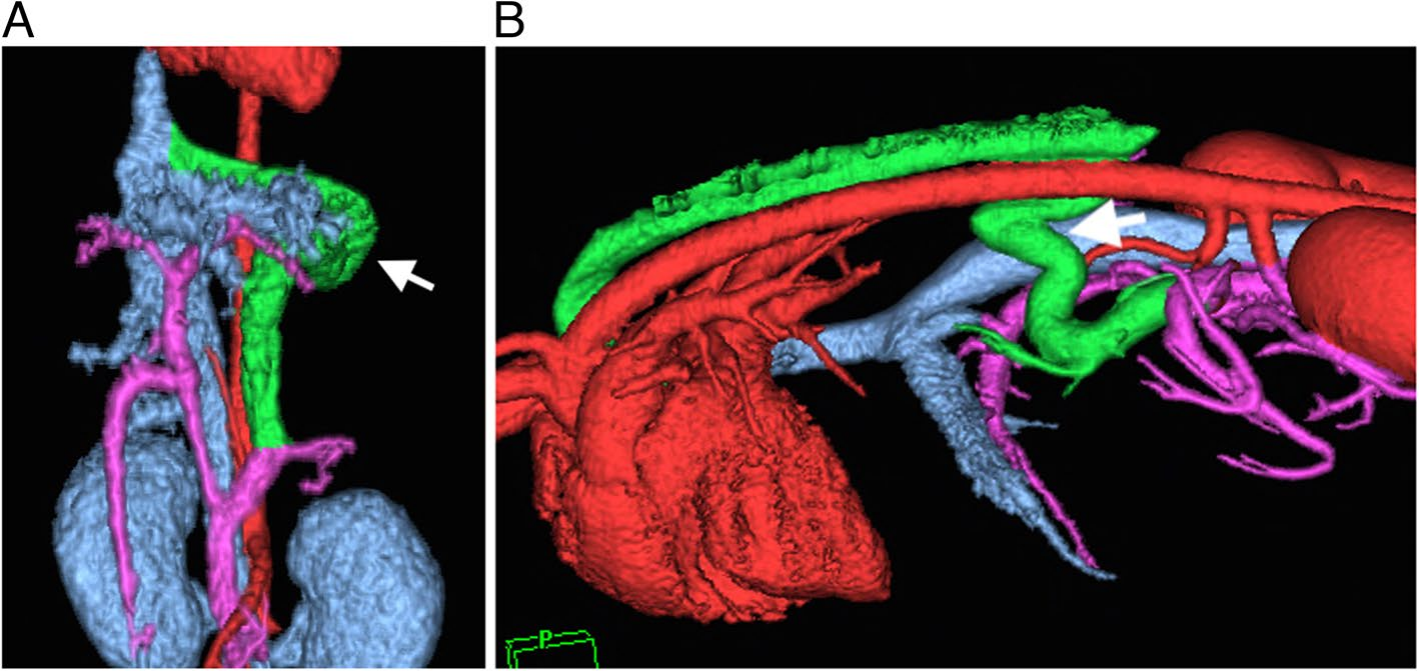
Three-dimensional computed tomography (CT) images of left gastrophrenic and gastroazygos PSS. **A** Ventrodorsal view of the left gastrophrenic PSS. Green color and white arrows indicate the left gastric vein, PSS, and phrenic vein (red = arteries and heart; blue = caudal vena cava, hepatic veins, and kidneys; purple = portal veins). Grade 2 intrahepatic portal vascularity was observed. **B** Lateral view of the left gastroazygos PSS. Green color indicates the left gastric vein, PSS, and azygos vein (red = arteries, heart, and kidneys; blue = caudal vena cava and hepatic veins; purple = portal veins). Grade 3 intrahepatic portal vascularity was observed. PSS: portosystemic shunt

The type of PSS and grade of intrahepatic portal vascularization were identified by computed tomography (CT) in all dogs as shown in Table 1 and Fig. 1A and B. The median grade of PSS by CT was grade 3 [1–4, *n* = 39]. Most dogs with left gastrophrenic and gastroazygos shunts were categorized in grade 3.

**Table 1:** Portosystemic shunt (PSS) type and grade classification of all dogs

PSS type	N	CT grading				
		Grade 1	Grade 2	Grade 3	Grade 4	N/A
Left gastrophrenic	30	1	7	11	9	2
Left gastroazygos	8	0	2	4	1	1
Left gastrocaval	1	0	1	0	0	0
Splenocaval	1	0	0	0	1	0
Splenophrenic	1	0	1	0	0	0
Left colocaval	1	0	1	0	0	0
Total	42	1	12	15	11	3

^a^*N/A* Non assessment

**Fig. 2:**
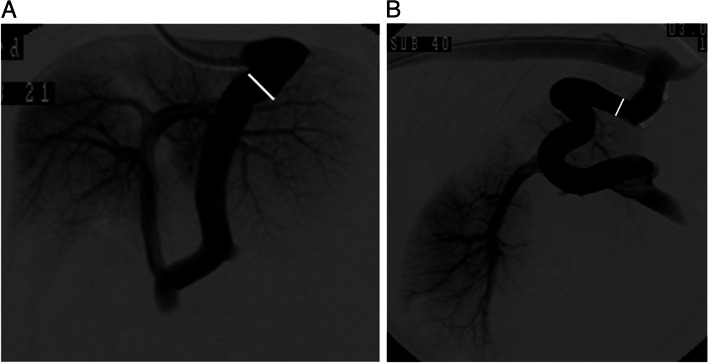
Transvenous retrograde portography (TRP) images of left gastrophrenic and gastroazygos PSS. Images were acquired in the digital subtraction angiography (DSA) mode. During balloon inflation, the contrast agent was injected via the balloon-tipped multipurpose catheter, which was advanced into the PSS. The PSS diameter was measured according to on the (**A**) ventrodorsal view of the left gastrophrenic PSS and (**B**) right lateral view of the left gastroazygos PS. White line shows the site at which the PSS diameter was measured. PSS: portosystemic shunt

**Fig. 3:**
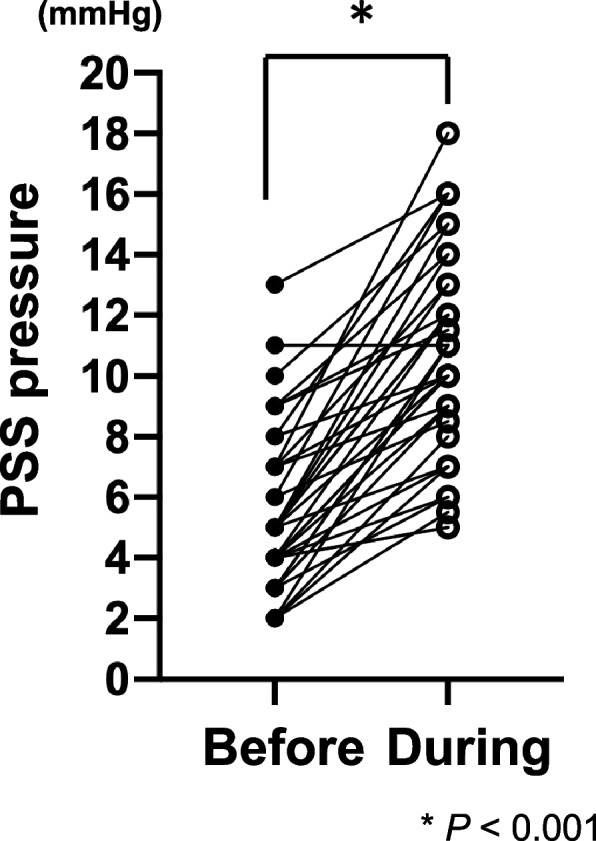
Pressure in the PSS before and during temporal occlusion of the PSS. The PSS pressure during the temporal occlusion was significantly higher than that before. Before = PSS pressure before balloon inflation; during = PSS pressure during balloon inflation. *p < 0.001. PSS: portosystemic shunt

Percutaneous jugular venous access was possible in all the dogs. As per the transvenous retrograde portography (TRP) findings, the median diameter at the site of coil implantation was 5.5 mm [2.5–8.7 mm]. During TRP, intrahepatic portal vascularity was clearly detectable in all dogs (Fig. 2 A, B; Additional files 1 and 2). The median values of blood pressure in the PSS (pPSS) before and during temporal occlusion by balloon inflation were 4.8 mmHg [2.0–13.0 mmHg] and 8.6 mmHg [5.0–18.0 mmHg], respectively (Fig. 3). The value of pPSS was significantly higher during temporal occlusion than before (*p* < 0.001); the median increase rate of pPSS was 2.2 [1.0–5.5].

The embolization coils were implanted with no immediate migration in all dogs (Additional files 3 and 4). The numbers of MReye® embolization coils implanted were one in 4 dogs, two coils in 24 dogs, three in seven dogs, four in 1 dog, and five in 1 dog. In contrast, one Interlock™-35 coil was implanted in five dogs each. The median maximum diameters of the implanted MReye® embolization coils (COOK Inc., Bloomington) and Interlock™-35 coils (Boston Scientific, Marlboro) were 8.0 mm [4.0–12.0 mm] and 6.0 mm [4.0–8.0 mm], respectively. The median ratio of the maximum size of implanted coils to the PSS diameter was 1.3 [1.1–2.2]. The median time from insertion to removal of the introducer sheath (PTCE time) was 40 min [23–75 min] in 40 dogs. In the first two dogs, the description of PTCE time was unclear in the medical records because laparoscopic ovariohysterectomy was subsequently performed. The median time of fluoroscopic irradiation was 9 min [4–26 min] in the 31 dogs for whom it had been recorded. No intraoperative complications, including hemorrhage or coil migration, were observed in any of the dogs. In addition, PTCE did not cause mortality.

Postoperatively, no dogs developed hematoma in the cervical area after removal of the introducer sheath. All dogs were in good general condition until discharge, but some showed gastrointestinal signs such as vomiting and diarrhea in three and two dogs, respectively. The median hospitalization period was 5.1 days [2–20 days]. No clinical signs associated with post-attenuation neurological signs were observed after PTCE in any of the dogs.

**Fig. 4:**
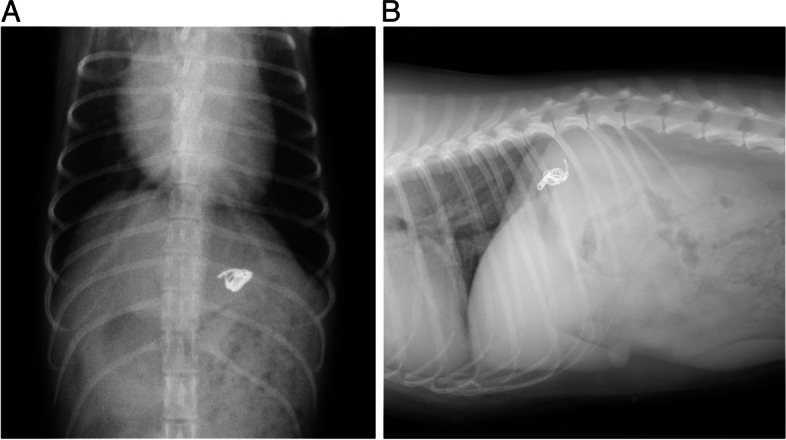
Radiographic images after percutaneous transvenous coil embolization (PTCE). **A** Ventrodorsal view: two 6-mm embolization coils were implanted into the left gastrophrenic PSS. **B** Right lateral view: two 10-mm and one 8-mm embolization coils were implanted into the left gastroazygos PSS. PSS: portosystemic shunt

The median postoperative follow-up time was 5.5 months [0.5–32 months]. The median fasting and postprandial TBA concentrations significantly decreased to 8.2 μmol/L [0.3–45.1 μmol/L, *n* = 38, *p* = 0.0028] and 19.8 μmol/L [0.3–106.7 μmol/L, *n* = 38, *p* = 0.0018], respectively, approximately one month after PTCE. At the postoperative follow-up, three dogs (#1, #2, and #3) showed elevated fasting and postprandial TBA concentrations (#1, fasting: 59.3 μmol/L, postprandial: 111.8 μmol/L; #2, fasting: 38.9 μmol/L, postprandial: 108.8 μmol/L; #3, fasting: 88.1 μmol/L, postprandial: 35.5 μmol/L). In these dogs, the intraoperative values of pPSS before and during balloon dilation were 11 and 11 mmHg, respectively, in dog #1; 3 and 8 mmHg, respectively, in dog #2; and 5 and 14 mmHg, respectively, in dog #3. Follow-up CT was performed postoperatively in these dogs. In dog #1, complete closure of PSS was confirmed by postoperative CT, and no collateral vessels or small residual PSS were detected. However, the other two dogs had residual PSS flow. One dog underwent ameroid constrictor placement approximately 4 months after PTCE, and another dog underwent surgical ligation approximately 4 years after PTCE. The portal pressure at the time of occlusion in the operated cases was 15 mmHg for ameroid constrictor placement and 14 mmHg for ligation. In the remaining 40 dogs (95.2%), coil migration was not observed on abdominal radiography in every consultation day since the PTCE procedure (Fig. 4 A, B).

## Discussion

Three previous studies have reported the use of coil embolization for treating single extrahepatic PSS in a total of eight dogs [17, 19, 20]. After coil embolization, the clinical signs associated with PSS resolve in four of these eight dogs. Among the remaining four dogs (50%), two died because of coil migration to the level of the main pulmonary artery, one was euthanized because of acute portal hypertension, and another was euthanized because of significantly decreased liver function. Thus, the outcome of coil embolization for the treatment of canine extrahepatic PSS has been far from satisfactory for veterinarians and pet owners. On the other hand, a previous study, using a modified Amplatzer vascular plug (AVP) in place of embolization coils, reported that the modified AVP was placed in six of seven dogs with an extrahepatic PSS and resulted in complete occlusion in five of the six dogs [21]. Our study showed that PTCE was feasible in small-breed dogs (lowest body weight 1.4 kg) and resulted in a clinical success rate of 95.2%, with a decrease in TBA levels to the normal range as well as improvements in clinical signs. In addition, at the middle-term follow-up, no dogs died in our study.

In dogs with single extrahepatic PSS, case selection is considered to be important for successful PTCE treatment. Body weight may be a limiting factor for PTCE because catheterization is difficult in small-sized dogs. A previous study reported coil migration in two dogs weighing less than 3 kg [19]. In our study, nine dogs weighed less than 3 kg, and no coil migration was observed in any of them. Furthermore, even in dogs weighing 1.4 kg, no complications associated with PTCE were observed. In our study, the largest PSS diameter included was 8.7 mm, because 9 mm was the upper limit to be able to place 12 mm coils, the largest coils that were used. In addition, large embolization coils are not available in the market. To prevent coil migration, the diameter of the implanted coils must be slightly greater than the diameter of the PSS. The median ratio of the maximum diameter of implanted coils to the measurable diameter of PSS by TRP was approximately 1.3 in our study; thus, there were no cases of coil migration. For coil embolization in intrahepatic PSS, a caval stent is generally used to prevent coil migration [20, 22]. Likewise, implantation of a caval stent may be effective in dogs with extrahepatic PSS. However, the CVC is small, and it is difficult to choose a suitable stent size in small dogs and cats with extrahepatic PSS. Although stent implantation was not performed in our study, coil migration did not occur in any of the dogs. The implanted coils that were approximately 1.3 times the vessel size may be immovable in the PSS. In addition, stent implantation into the azygos vein is technically difficult. Therefore, our study results suggest that coil migration was prevented by the suitably sized coils in small dogs with extrahepatic PSS. In addition, our procedure for PTCE has the advantage of lower cost because a caval stent was not applied. However, the maximum coil diameter was 2.2 times the shunt vessel diameter in our study. In case that the diameter of coil is substantially larger than that of PSS, the coils may not be well-rounded and implanted in the intended position, which might also increase the risk of coil migration. Therefore, the choice of coils with appropriate diameter is essential for preventing the coil migration. However, as for the numbers and size of which coils are required for the suitable PTCE, the present study could conclude no clear definitive standardization. The further investigations are required to clarify the suitable size of coils for PTCE without a caval stent in canine extrahepatic PSS.

In the present study, acute portal hypertension did not occur in any dog after PTCE. Portal hypertension may be caused by acute complete occlusion of the PSS after complete suture ligation in the presence of poor intrahepatic portal vascularity. All cases in this study had a clear intrahepatic portal branch based on TRPs. In the TRP prior to PTCE, the pPSS during the temporary occlusion of the PSS through balloon inflation increased, but it was < 18 mmHg in all dogs, and the increase rate of pPSS was approximately 2.2. If open surgery is chosen, complete suture ligation might be feasible, as the maximum limit of portal pressure for complete ligation is 15–18 mmHg [23]. However, PTCE has the advantages of short operation time and minimum pain with no incisions. The present study could not demonstrate that PTCE was effective in cases of significant portal hypertension caused by balloon inflation in TRP. In the future, further investigations are needed to clarify the indications and usefulness of PTCE for the treatment of canine extrahepatic PSS with significant portal hypertension caused by balloon inflation.

In general, PSS attenuation by coil embolization occurs gradually. However, the rate of PSS attenuation may depend on the number and size of the implanted coils. A previous study reported acute portal hypertension after coil embolization in one dog [19]. In our study, preoperative CT angiography was used to evaluate intrahepatic portal vascularity; however, intrahepatic portal vascularity grades varied between 1 and 4 in our study. Complete surgical ligation may be possible in dogs with grade 1 intrahepatic portal vascularity, based on CT findings that can have a major impact on the choice of treatment for canine extrahepatic PSS. However, the major difference between CT and TRP was obstruction of the PSS by balloon inflation; thus, intrahepatic portal vascularity might be more accurately evaluated by TRP than by CT. It is preferable to evaluate intrahepatic portal vasculature using TRP instead of preoperative CT to estimate the risk of acute portal hypertension after coil implantation. Preoperative CT is essential for obtaining morphological information, as well as for making a definitive diagnosis of PSS. Based on the CT images, the diameter and length of the PSS can be determined; however, more research is needed to determine the usefulness of these data. On the other hand, TRP not only provides information on intrahepatic portal vascularity but also causes a rise in portal pressure after complete occlusion of PSS. The pPSS measured in our study was considered to be equal to the portal pressure because the PSS communicates to the portal vein. The increase rate of pPSS during balloon inflation was approximately 2.2, and pPSS during balloon inflation did not increase over 18 mmHg, which is considered to be the upper limit of the increase in portal pressure from the standpoint of complete PSS attenuation by surgical ligation. We strongly believe that TRP prior to PTCE is useful for evaluating the feasibility of coil implantation and the chances of therapeutic success.

In the portoazygos shunt, the decision of coil implantation site is important for successful PTCE. However, the morphology of portoazygos shunt widely varies, so the advancement of the catheter from the azygos vein to shunt may be a little bit difficult in some cases. In our study, the advancement into the shunt has been success in all portoazygos shunts. Through those experiences, the preoperative CT might become an useful guide for the advancing the catheter and guidewire in the azygos vein and shunt. However, the further studies are required to decide the implantation site of portoazygos shunt in clinical settings.

CT was performed for evaluating the cause of high TBA levels after PTCE in the three dogs; it was found that one dog had no residual PSS flow, and the TBA concentrations subsequently tended to gradually decrease over time. Although serum TBA analysis is clinically useful for the diagnosis of canine PSS, it cannot indicate the type, severity, and shunt ratio of PSS. Serum TBA levels are also affected by impaired liver function; therefore, a combination of imaging modalities is necessary for the estimation of PSS closure [24]. In our clinical study, in all dogs with a significant decrease in postoperative TBA concentrations, the clinical signs and liver function, based on blood test data, showed improvements on postoperative evaluations; therefore, CT was not performed again because of the cost and requirement of general anesthesia. In three dogs with postoperative elevated TBA concentrations, complete PSS closure was confirmed by postoperative CT, which might not have detected collateral or small residual PSS. In addition, the implanted coils may cause artifacts in the image quality of CT after PTCE. Therefore, further investigations using CT are required to clarify the attenuation status of PSS after PTCE.

Being a technically demanding procedure, PTCE has a learning curve. The operative time decreased with an increase in the operator’s experience (data not shown). Furthermore, PTCE is believed to be less invasive than conventional open surgery, which includes surgical ligation, ameroid constrictor placement, and thin film banding, owing to the short anesthesia and operation times and absence of skin or abdominal incisions. Similar to conventional open surgery, PTCE may lead to good clinical outcomes in dogs with single extrahepatic PSS. Further large-scale clinical studies on PTCE are needed to confirm whether it can be used as the treatment of choice for canine single extrahepatic PSS. In addition, the establishment of veterinary training facilities for interventional radiology, including PTCE, is required.

A notable disadvantage of PTCE compared with conventional open surgery is the need for open surgery when performing cystotomy and neutering; however, the most significant disadvantage is its cost. It requires an X-ray fluoroscope equipped with digital subtraction angiography (DSA) capability and a radiolucent operation table. A large variety of catheters and embolization coils are also required. However, the number and types of equipment required for PTCE can be reduced; the minimal requirements include a 5-Fr introducer sheath, 5-Fr dual-lumen balloon-tipped multipurpose catheter, and 0.035-inch guide wire. In our study, two MReye® embolization coils or one Interlock™-35 coil were implanted in almost all dogs, all of which enabled effective PSS attenuation. Therefore, the cost of PTCE for the treatment of single extrahepatic PSS in dogs can be decreased. However, the cost of PTCE may remain higher than that of conventional open surgery. Affordable equipment and modalities should be developed and made commercially available in the future.

## Conclusion

Our study demonstrated that PTCE is clinically effective for the treatment of canine single extrahepatic PSS, with a treatment success rate of 95.2%. Therefore, PTCE is suggested to be a promising alternative to conventional surgical procedures for single extrahepatic PSS without portal hypertension in dogs.

## Methods

### Animals

Privately owned dogs with extrahepatic PSS treated by PTCE between April 2004 and October 2020 were included in the study. In all dogs, preoperative clinical signs associated with PSS were recorded, and fasting and postprandial TBA levels were measured preoperatively. A definitive diagnosis of extrahepatic PSS was made using CT findings.

### Computed tomography

CT scans of all dogs were performed using a 16 multidetector CT scanner (Aquilion 16, Canon Medical Systems Co., Otawara, Japan) or a 320 multidetector CT scanner (Aquilion ONE; Canon Medical Systems Co.). All helical scans started from the tip of the iliac wing, ended at the thoracic inlet, and proceeded in a cranial direction. Iohexol (Ioverin 300; Teva Pharma Japan Inc., Nagoya, Japan) was used as a contrast medium and administered at a dose of 2.5 mL/kg (750 mg/kg) via the cephalic vein with a power injector (Auto Enhance A-60; Nemoto-Kyorindo, Tokyo, Japan). The injection time was 15 s (injection speed, 3 mL/s). In dogs in whom the injection speed was calculated to exceed 3 mL/s, the injection time of the contrast medium would have to be within 20 s; therefore, the injection speed was set at 3 mL/s. Triple-phase CT scans were performed, and portal venous phase (20 s after the start of the arterial phase) images were used to diagnose PSS. For each dog, the PSS type was determined using the CT findings. Intrahepatic portal vascularity was based on a previously reported portographic grading system [25]. The grading was as follows: grade 1, no intrahepatic portal vasculature visible; grade 2, faint opacification of vestigial portal vessels; grade 3, faint opacification of a few second- or third-generation portal vessels; and grade 4, substantial opacification of third- and fourth-generation portal vessels.

### Percutaneous transvenous coil embolization

Each dog was premedicated with atropine sulphate (0.04 mg/kg; Nipro ES Pharma Co., Settsu, Japan) subcutaneously and intubated after induction with intravenous propofol (Mylan, Mylan Seiyaku Ltd., Tokyo, Japan). General anesthesia was maintained with isoflurane (1.5–2%; IsoFlo, Zoetis Japan, Tokyo, Japan), vaporized in oxygen and continuous infusion of remifentanil (20–40 μg/kg/h; Ultiva, Janssen Pharmaceutical K.K. Tokyo, Japan). Dogs with portoazygos shunts were positioned in right-sided lateral recumbency, while the others were operated upon in dorsal recumbency. Using a C-arm fluoroscope (SIREMOBIL Compact, Siemens Co, Berlin, Germany; BV Pulsera, Phillips Co, Amsterdam, Netherlands) equipped with DSA capability, a sizing catheter (COOK Inc, Bloomington, IN, USA) was inserted into the esophagus. Fluoroscopic images of the sizing catheter were acquired and saved as references. A 5-Fr introducer sheath (Cook Inc., Bloomington) was percutaneously inserted into the left jugular vein in dogs with portoazygos shunts and into the right jugular vein in dogs with portocaval or portophrenic shunts. A 5-Fr dual-lumen balloon-tipped multipurpose catheter (Terumo Clinical Supply Co., Kagamihara, Japan) with a balloon diameter of 9 mm, when dilated to maximum capacity, was inserted into the sheath. The balloon-tipped catheter was directly advanced into the PSS using a 0.035-inch guide wire (COOK Inc., Bloomington) under fluoroscopic guidance while comparing with the CT angiographic findings.

The pPSS prior to temporal occlusion was measured using a balloon-tipped catheter. The balloon was then inflated with a diluted contrast medium, and the pPSS during temporal occlusion by balloon inflation was measured. After both pressures were measured, TRP was performed during balloon inflation. The contrast medium was injected through the catheter at a dosage of 1–2 mL/kg manually, with continuous fluoroscopic evaluation and DSA recording. After TRP revealed that the tip of the catheter was advanced into the PSS with the reference to the CT angiographic images, the diameter of the PSS was measured at a position about 5–10 mm further into the shunt vessel from the catheter tip. The inflated balloon was released after completion of the TRP.

For the PTCE, 0.035-inch MReye® embolization coils (COOK Inc., Bloomington) or Interlock™-35 Coil Fibered IDC™ Occlusion system (Boston Scientific, Marlboro, MA, USA) were used. Our facility could easily purchase coils sized < 12 mm, and the size of implanted coils was set to be approximately 1.3 times the size of PSS. In addition, the maximum diameter of the balloon was 9 mm, owing to its inflation in the 5-Fr balloon catheter for TRP. Therefore, the maximum diameter of PSS to be included for PTCE was 9 mm. The coils were pushed and implanted using a 0.035-inch guidewire through the balloon-tipped catheter while its tip was maintained in the PSS. The coils were implanted in the same position where the diameter of the PSS was measured. TRP and PTCE in all dogs were performed by one veterinarian (K. A.). Fluoroscopic irradiation time was recorded for all dogs. In addition, the operation time, considered as the interval between the sheath placement and removal, was also recorded.

### Postoperative evaluation and follow-up

Postoperative clinical signs and intra- and postoperative complications were evaluated. Coil migration was assessed by abdominal radiography. When the clinical signs and blood test findings, including TBA concentrations, did not improve at the time of the postoperative follow-up examination, CT was performed to evaluate the residual PSS flow. To stop residual flow, a second surgical intervention was performed.

The dogs were re-examined 1–2 weeks after discharge, and the physical examination, blood test and radiography were performed for up to 1 year after surgery as needed. Furthermore, fasting and postprandial TBA measurements were recorded during follow-up from 2 weeks to 1 year postoperatively, although the duration of the measurements varied from case-to-case. The dogs with elevated TBA in the follow-up period underwent CT again to check for residual shunt flow or the occurrence of multiple acquired shunts.

### Statistical analysis

The relative increase ratio of pPSS was calculated as follows: the increase ratio of pPSS = pPSS during temporary occlusion/pPSS before temporary occlusion. Wilcoxon matched-pairs signed rank test was used for comparison of the pPSS before and during the temporary occlusion, and fasting and postprandial TBAs, before and after the PTCE, were analyzed. Statistical significance was set at *P* < 0.05.

### Supplementary Information

**Additional file 1.** Movie of transvenous retrograde portography (TRP) with digital subtraction angiography (DSA) of the left gastrophrenic shunt (LGP). During balloon dilation, the contrast agent was injected via the balloon-tipped multipurpose catheter, which was advanced into the shunt vessel. This movie facilitates the identification of LGP and intrahepatic portal venous branches.

**Additional file 2.** Movie of transvenous retrograde portography (TRP) with digital subtraction angiography (DSA) of the left gastroazygos shunt (LGA). During balloon dilation, the contrast agent was injected via the balloon-tipped multipurpose catheter, which was advanced into the shunt vessel. This movie facilitates the identification of LGA and intrahepatic portal venous branches.

**Additional file 3.** Movie of coil implantation for a left gastrophrenic shunt. In this case, 0.035-inch MReye® embolization coils (COOK Inc., Bloomington, IN, USA) were used.

**Additional file 4.** Movie of coil implantation for left gastroazygos shunt. In this case, 0.035-inch MReye® embolization coils (COOK Inc., Bloomington, IN, USA) were used.

**Additional file 5: Supplementary Table.** Diameter of shunt vessel and number and type of implanted coils.

## Data Availability

All data supporting the conclusions of this article are included within the article.

## Abbreviations

AVP: Amplatzer vascular plug
CT: Computed tomography
DSA: Digital subtraction angiography
PSS: Portosystemic shunt
pPSS: Blood pressure in the PSS
PTCE: Percutaneous transvenous coil embolization
TBA: Serum total bill acid concentrations
TRP: Transvenous retrograde portography
